# Endothelial nitric oxide synthase deficiency exacerbates SARS‐CoV‐2 induced neuropathology in a mouse model of vascular dementia

**DOI:** 10.1002/alz.092618

**Published:** 2025-01-03

**Authors:** Saifudeen Ismael, Meenakshi Umar, Gregory J Bix

**Affiliations:** ^1^ Department of Neurosurgery, Clinical Neuroscience Research Center, Tulane University School of Medicine, New Orleans, LA USA; ^2^ Department of Neurology, Tulane University School of Medicine, New Orleans, LA USA

## Abstract

**Background:**

SARS‐CoV‐2 causes a variety of neurological sequelae in COVID‐19 survivors, including fatigue and cognitive dysfunction. Endothelial dysfunction is the unifying and central mechanism of COVID‐19 illness and a major risk factor for vascular dementia (VaD). Endothelial dysfunction stems, in part, from an imbalance between nitric oxide (NO) generated by the endothelial nitric oxide synthase (eNOS) and reactive oxidant species produced by uncoupled‐eNOS. Clinical observations suggest that *decreased NO bioavailability appears as a likely pathogenic factor of endothelial dysfunction in COVID‐19 patients*. We hypothesize that eNOS deficiency contributes to brain endothelial dysfunction in SARS‐CoV‐2 infection and SARS CoV‐2 infection promotes early onset VaD in eNOS deficient mice.

**Methods:**

6‐month‐old WT/eNOS± male mice were infected with 1 × 10^4^‐pfu mouse‐adapted (MA10) SARS‐CoV‐2 intranasally and animals were evaluated acutely out to 3 days‐post‐infection (3dpi) for changes in body weight and clinical signs of illness. Genomic and sub‐genomic viral copy numbers were also quantified. RNA was isolated whole‐brain and was analyzed for quantitative PCR and immunofluorescence for markers of inflammation and blood‐brain barrier permeability.

**Results:**

MA10 infection induced significant weight loss in WT and eNOS± mice. eNOS± mice exhibited more disease‐associated weight loss (∼15%) than WT‐infected mice, but no infection‐induced mortality was observed in either group. We then confirmed MA10 viral copy in the lungs of infected, but not uninfected, WT MA10 and eNOS± mice at 3 dpi (noted to be similar in both groups). However, we did not detect the virus in the brains of the MA10‐infected WT and eNOS± mice. Real‐time PCR analysis of mouse whole brain‐isolated mRNA showed that the proinflammatory mediators CCL2 and IL‐6 were elevated in both the WT and eNOS^±^ mice (eNOS^±^ > WT) following MA10 infection. Immunofluorescent analysis showed increased Iba1 (microglia marker) fluorescent intensity in the cortex of eNOS^±^ mice following MA10 infection than WT mice (mock or MA10 infected) and eNOS^±^ mock mice.

**Conclusions:**

The eNOS± mice experience worsened acute SARS‐CoV‐2‐associated morbidity indicated by increased weight loss, glial activation, and neuroinflammation than WT mice despite a similar pulmonary viral load. This is the first experimental evidence investigating a link between eNOS and neuropathology associated with SARS‐CoV‐2.

